# Development of a Genomic Signatures-Based Predictor of Initial Platinum-Resistance in Advanced High-Grade Serous Ovarian Cancer Patients

**DOI:** 10.3389/fonc.2020.625866

**Published:** 2021-03-05

**Authors:** Yuan Li, Xiaolan Zhang, Yan Gao, Chunliang Shang, Bo Yu, Tongxia Wang, Junyan Su, Cuiyu Huang, Yu Wu, Hongyan Guo, Chunfang Ha

**Affiliations:** ^1^ Department of Obstetrics and Gynecology, Peking University Third Hospital, Beijing, China; ^2^ Lifehealthcare Clinical Laboratories, Hangzhou, China; ^3^ Department of Gynecology and Obstetrics Department, General Hospital of Ningxia Medical University, Yinchuan, China

**Keywords:** ovarian cancer, initial platinum resistance, DNA damage repair, homologous recombination deficiency, BRCA

## Abstract

**Background:**

High grade serous ovarian cancer (HGSOC) is the most common subtype of ovarian cancer. Although platinum-based chemotherapy has been the cornerstone for HGSOC treatment, nearly 25% of patients would have less than 6 months of interval since the last platinum chemotherapy, referred to as platinum-resistance. Currently, no precise tools to predict platinum resistance have been developed yet.

**Methods:**

Ninety-nine HGSOC patients, who have finished cytoreductive surgery and platinum-based chemotherapy in Peking University Third Hospital from 2018 to 2019, were enrolled. Whole-genome sequencing (WGS) and whole-exome sequencing (WES) were performed on the collected tumor tissue samples to establish a platinum-resistance predictor in a discovery cohort of 57 patients, and further validated in another 42 HGSOC patients.

**Results:**

A high prevalence of alterations in DNA damage repair (DDR) pathway, including *BRCA1/2*, was identified both in the platinum-sensitive and resistant HGSOC patients. Compared with the resistant subgroup, there was a trend of higher prevalence of homologous recombination deficiency (HRD) in the platinum-sensitive subgroup (78.95% vs. 47.37%, p=0.0646). Based on the HRD score, microhomology insertions and deletions (MHID), copy number changes load, duplication load of 1–100 kb, single nucleotide variants load, and eight other mutational signatures, a combined predictor of platinum-resistance, named as DRDscore, was established. DRDscore outperformed in predicting the platinum-sensitivity than the previously reported biomarkers with a predictive accuracy of 0.860 at a threshold of 0.7584. The predictive performance of DRDscore was validated in an independent cohort of 42 HGSOC patients with a sensitivity of 90.9%.

**Conclusions:**

A multi-genomic signature-based analysis enabled the prediction of initial platinum resistance in advanced HGSOC patients, which may serve as a novel assessment of platinum resistance, provide therapeutic guidance, and merit further validation.

## Introduction

Epithelial ovarian cancer (EOC) is the malignant carcinoma with the highest mortality in women worldwide, which led to an annual death of 14,070 and 30,886 cases in the United States and China, respectively (1). High grade serous ovarian cancer (HGSOC), which accounts for 70%~80% of ovarian carcinoma-associated death, is conventionally treated with surgery and chemotherapy, including paclitaxel and carboplatin (2, 3). However, nearly 20%–40% of patients would not response to the initial platinum-based therapy (4). Primary resistance to platinum therapy poses a severe challenge to the treatment of advanced ovarian cancer (5, 6). A large decrease of median survival time to less than 2 years is observed in platinum-resistant HGSOC patients, and less than 15% of them would response to subsequent chemotherapy (7).

Platinum is demonstrated to induce extensive DNA damage by inducing DNA cross-links, thereby suppressing tumor cells proliferation and enhance the apoptosis proliferating cells, which leads to tumor eradication. Increasing evidence implicates that homologous recombination (HR) repair system might mediate the resistance to platin-induced cross-links break (8). Approximately, 50% of HGSOC have a homologous recombination deficiency (HRD) mainly due to genetic and epigenetic changes in HR pathway genes. Primary resistance to platinum is found to be associated with diverse biological processes, including the genetic mutation, alterations of anti-apoptosis signaling pathways, expression of neoplastic antigens, and abnormal DNA damages repair. A better understanding of drug-resistant mechanisms is required to design strategies to predict potential drug resistance and improve clinical outcomes.

Serval biomarkers of immune changes, epigenetics, and genomics alterations in DNA repair systems have been reported to be correlated with platinum-sensitivity of HGSOC. *BRCA1/2* mutations were the most common alterations, which accounted for nearly 22.6% of HGSOCs, while somatic *BRCA1/2* mutations were found in 6% to 7% HGSOCs (9, 10). Additionally, defective HR system is an essential target in EOC, which predicts the therapeutic efficacy of platinum analogues and PARP inhibitors in this disease (11). HRD and absence of CCNE1 amplification are associated with improved survival of ovarian cancer patients treated with platinum (12). Intriguingly, HRD, based on three independent measures of genomic instability, including telomeric allelic imbalance (TAI), large-scale state transition (LST) and loss of heterozygosity (LOH), provides a better evaluation of HR function loss in predicting the response to DNA-damaging agents in ovarian cancer (13, 14). Though the majority patients with *BRCA1/2* and/or HRD are platinum-sensitive, the overlap is still limited (15). Other genetic features, including gene expression, gene variants, single nucleotide polymorphism and copy number changes, have been studied in the value of predicting of platinum sensitivity in ovarian cancer (16–18). Based on the large TCGA dataset (The Cancer Genome Atlas), Yin et al. devised a 131-gene signature associated with platinum resistance, but was still not applicable to clinical use (19). By utilizing the TCGA data, a classifier based the expression of 23 DNA repair genes was found, which was better than the standard clinical feature in predicting the response of platinum therapy (0.65 vs. 0.52) (20). Furthermore, a large systematic review of 42 studies, which explore molecular signatures for predicting platinum-resistance in ovarian cancer, showed that the genetic signatures were inconsistent among these studies and none of them are currently applicable to clinical use (21). Despite several prediction models of platinum resistance reported, the results are mainly preliminary and there are still no relatively accurate clinical tools to predict the response to platinum in HGSOC therapy.

In our study, we developed a multi-genetic signatures-based analysis using the whole genome and exome sequencing. Both WES and WGS data on 57 primary tissues from HGSOC patients were used to perform a comprehensive analysis of multiple DNA damages associated factors, including HRD, MHID, TMB, mutational signatures, and number of neoantigens for their correlation with the platinum sensitivity in HGSOC patients. The performance of the signature was validated in an independent cohort of 42 HGSOC patients.

## Materials and Methods

### Study Population

Ninety-nine patients pathologically confirmed with HGSOC in Peking University Third Hospital were enrolled in this study, consisting of 57 and 42 samples for the discovery and validation cohort, respectively. All patients had finished surgery and the first-line platinum-based therapy form 2018 and 2019. The Ethical approval was obtained from Ethics Committee of Peking University Third Hospital and all patients had provided a written informed consent. Formalin-fixed, paraffine-embedded (FFPE) slides of each patient’s primary tumor tissue before chemotherapy were collected and underwent the following analysis. Patients were classified into platinum-sensitive and platinum-resistant subgroups according to the consensus statement of Gynecologic Cancer Intergroup (GCIG) (22).

### DNA Extraction

DNA from the FFPE samples was extracted using DNeasy Blood & Tissue Kit (Qiagen, Inc.) according to the manufacturer’s instructions. The purified DNA was quantified using the Qubit 3.0 Fluorometer (Life Technologies, Inc.) and their quality was evaluated by a PCR assay on StepOnePlus System (Life Technologies, Inc.). The Accel-NGS 2S HYB DNA LIBRARY KIT (Swift Biosciences, 23096) and HotStart ReadyMix (KAPA, KK2612) were used for library preparation and amplification, respectively. The amplified libraries were purified by SPRI SELECT (Beckman, B23319).

### Whole Genome Sequencing

Each sample’s library underwent paired-end sequencing on a NovaSeq 6000 platform (Illumina) with a 150 bp read length. Then mean depth of sequencing for each sample was 30×.

### Whole Exome Sequencing

The amplified libraries were captured with xGen Exome Research Panel v2 (IDT), whose target region was 33 Mb. Finally, samples underwent paired-end sequencing on a Novaseq 6000 platform (Illumina) with a 150 bp read length. The mean depth of sequencing for each sample was 500×.

### The Homologous Recombination Deficiency Analysis

The homologous recombination deficiency score (HRD score), which was depicted as genomic instability score or genomic scar score in some research, was calculated using a scarHRD R package (23). Each HRD score was calculated as described in previous reports, in which a combination of the numbers of loss-of-heterozygosity, large scale transitions and telomeric allelic imbalances in the whole genome were used (24). The value of 42 was defined as the threshold of high HRD score (25).

### Mutation Analysis

Raw sequencing data generated in the WES were aligned to the reference human genome (UCSC hg19) through Burrows-Wheeler Aligner and producing a binary alignment/map (BAM) file. After the duplicate removal and local realignment, the Genome Analysis Toolkit (GATK) was used for calling single nucleotide variation (SNV) and short insertions/deletions (indels). Variants were annotated using the ANNOVAR software tool. Variants with allele frequency beyond 1% in each sample were selected and further annotated according to the Catalog of Somatic Mutations in Cancer (COSMIC) database. The functional classification of each mutation was followed the interpretation, reporting standards and guidelines recommended by the Association for Molecular Pathology, American Society of Clinical Oncology, and College of American Pathologists (ASCO/CAP) (26).

### Tumor Mutation Burden Analysis

The tumor mutation burden in the whole exome region of each sample was calculated according to a published and widely applied method (27).

### Mutational Signatures

We performed a supervised analysis of mutational signatures of the data in the WES with the R package YAPSA and computed a linear combination decomposition of the mutational catalogue with known and predefined signatures by non-negative least squares (NNLS). Mutational catalog correction was performed to account for differences in the occurrence of triplet motifs by comparing the whole genome to WES capture regions. We used a set of 30 publicly available mutational signatures AC1-AC30 (AC standing for Alexandrov COSMIC).

### Microhomology Insertion and Deletion

MHID in the whole exome was calculated using the software SigProfilerMatrixGenerator, which explored and visualized all types of small mutation events (including substitutions, insertions, deletions and doublet substitutions) (28).

### Mutational Signature Set Classifier

To train a platinum-sensitivity related model, we adopted a leave-one-study-out cross-validation approach and assessed performance of three algorithms (LASSO) by using an R wrapper package with all depicted genomic signatures in the WGS and WES data.

## Results

### Mutational Profiles of Platinum-Sensitive and Resistant HGSOC Patients

**Figure 1:**
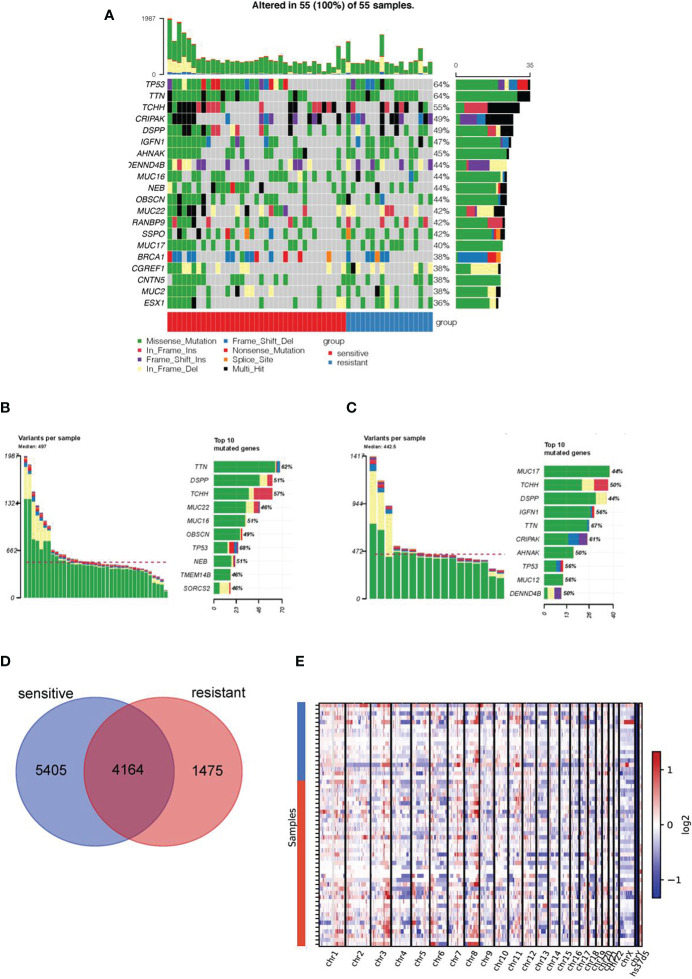
Mutation landscape in platinum-sensitive and resistant HGSOC patients. **(A)** Mutation landscape of top 20 genes in 55 HGSOC samples, including 37 platinum-sensitive and 18 resistant cases. The heat map shows the distribution of top 20 genes across samples, platinum response and mutational type information. The mutational profiles of the rest two patients were not shown in this figure due to the deficiency of alterations in these 20 genes. Specific distribution of top 10 genes alterations with different mutational types in in platinum-sensitive **(B)** and resistant patients **(C)**. **(D)** The Venn diagram showed the number of co-mutated and uniquely-mutated genes in the platinum sensitive and resistant HGSOC patients. **(E)** Copy number changes in platinum-sensitive and resistant patients.

Overall, a median number of 497 and 443 variants was found in the WES data of the platinum-sensitive and resistant subgroups, respectively. Among them, *TP53* (64%) and *TTN* (64%) were the most prevalent genes in all samples, which were also highly mutated in the HGSOC from The Cancer Genome Atlas Program (**Figure 1A**). Differences, but not significant, in the prevalent genes of platinum resistant and sensitive groups were found, including *MUC22*, *MUC16*, *OBSCN*, *NEB*, *TMEM14B*, *SORCS2*, *IGFN1*, *CPIPAK*, *AHNAK*, *MUC12*, and *DENND4B* (**Figures 1B, C** **)**.

Next, we investigated the difference of genetic alterations between the two subgroups. As shown in the Venn diagram, the two groups shared 4164 mutated genes, and 5405 and 1475 uniquely mutated genes were found in platinum-sensitive and resistant subgroup, respectively (**Figure 1D**). No significant difference in the copy number change in chromosome region was found between two groups (**Figure 1E**).

### Prevalence of *BRCA1/*2 and DNA Damage Repair (DDR) Related Alterations in Platinum Sensitive and Resistant HGSOC Patients

**Figure 2:**
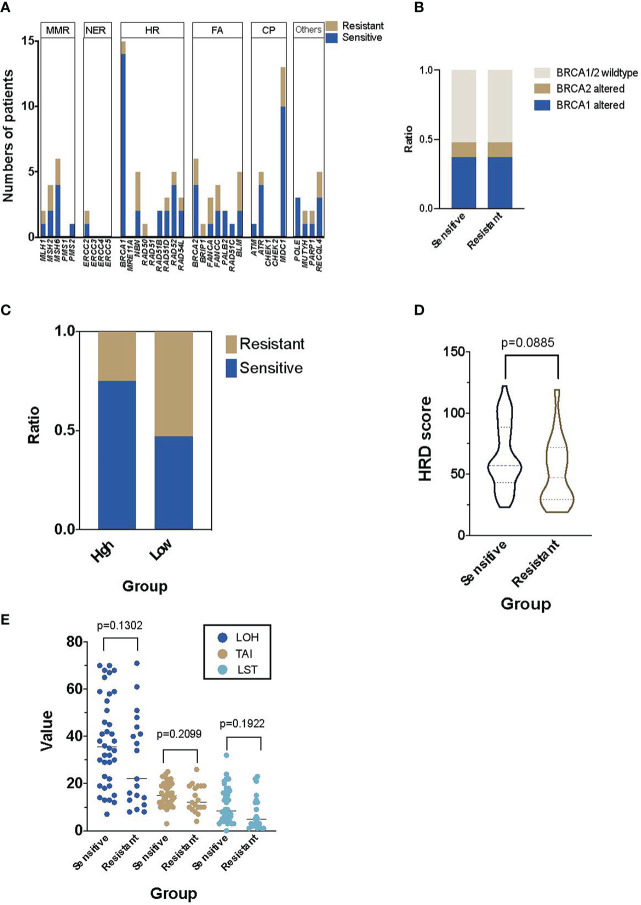
The prevalence of DNA damage repair (DDR) and homologous recombination deficiency (HRD) in the Platinum-sensitive and resistant HGSOC patients.**(A)** The distribution and numbers of HGSOC patients in main DDR genes. **(B)** The distribution of patients with BRCA1/2 alterations in platinum-sensitive and resistant HGSOC patients. **(C)** The distribution of platinum-sensitive and resistant HGSOC patients in the high and low HRD score groups. **(D)** The difference of HRD score in platinum-sensitive and resistant HGSOC patients. **(E)** Details of the components of HRD score, including LOH, TAI and LST in platinum-sensitive and resistant HGSOC patients. LOH, loss-of-heterozygosity; TAI, telomeric allelic imbalances; LST, large scale transitions.

In the discovery cohort of 57 patients, 80.70% of them (46/57) had at least one alteration in the 34 candidate DDR genes, though most alterations had unknown function (29). No specific distribution of alterations in DDR pathway was identified (**Figure 2A**). Interestingly, the prevalence of *BRCA1/2* alterations was similar among platinum-sensitive (18/38) and resistant groups (9/19, **Figure 2B**).

### HRDscores in Platinum-Sensitive and Resistant Subgroups

In the platinum-sensitive subgroups, 30 of the 38 patients had a high HRDscore. Among them, 14 samples (36.84%) were HRD positive but without any *BRCA1/2* alteration. Compared with the resistant subgroup, there was an insignificant trend of higher prevalence of positive HRDscore in the sensitive subgroup (78.95% vs. 47.37%, p=0.0646, **Figure 2C**). However, no significant difference in the value of HRD score between platinum-sensitive (median 56, range 23–122) and resistant (median 47, range 19 to 119) subgroups were identified (p=0.0885, **Figure 2D**). The individual components of HRDscore, including LOH, TAI and LST were exhibited in **Figure 2E**.

### Other Genomic Signatures in Platinum-Sensitive and Resistant Subgroups

**Figure 3:**
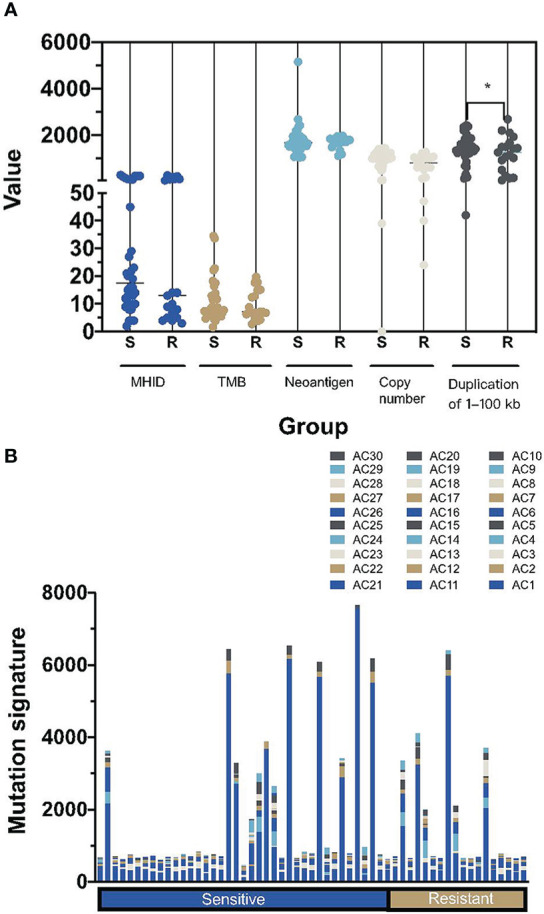
Summary of the other mutational signatures in platinum-sensitive and resistant HGSOC patients. **(A)** The difference of MHID, TMB, numbers of neoantigens, copy number changes and duplication load of 1–100 kb between platinum-sensitive and resistant HGSOC patients. **(B)** the distribution of mutational signatures, including AC1-AC30, in platinum-sensitive and resistant HGSOC patients. MHID, microhomology insertions and deletions; TMB, tumor mutation burden; S, sensitive; R, resistant. *p<0.05.

Next, we analyzed other genomic signatures revealed in the WES data, including MHID, TMB, neoantigen load, copy number, duplication load, and mutational signatures. Small insertions or deletions at DNA breakpoint joint, referred to as MHID, are the main feature of nonhomologous end joining (NHEJ)-mediated DNA double-strand break (DSB) repair. There was no significant difference in the MHID value between platinum-sensitive and resistant subgroups (median counts, 16 versus 13, p=0.77, **Figure 3A**). Similarly, the TMB, neoantigen load and copy number load were not significantly correlated to the platinum sensitivity individually. Notably, a significantly higher prevalence of duplication load of 1 to 100 bp was found in the platinum-sensitive subgroups (p=0.027).

Interestingly, we didn’t find a high prevalence of AC3, the only defective homologous recombination-based DNA damage repair signature defined in the COSMIC database, whether in the platinum-sensitive subgroups or in the *BRCA1/2* altered patients. Instead, a prevalence of AC1, associated with the deamination of 5-methylcytosine to thymine was detected in all HGOSC patients (**Figure 3B**).

### Multivariate Regression Determined a Platinum-Sensitivity Associated Predictor

We performed a lasso logistic regression model based on multiple genomic signatures in the 57 HGSOC patients. A new predictor, which was named DRDscore based on fourteen‐parameters, was constructed using the independent regression coefficients of each signature to predict the risk of platinum resistance. DRDscore was calculated as DRDscore=−0.00133(score of HRD) +0.000969 (score of MHID) +0.0003 (score of copy number) −0.00078 (score of Dup load 1–100 kb) −0.00024 (score of SNV load) +0.000013 (number of neoantigens)-0.000085 (score of AC1) +0.00046 (score of AC4) −0.0013(score of AC7) +0.00138(score of AC10) +0.00041 (score of AC12) +0.000678 (score of AC18) −0.00093 (score of AC20) −0.00081356 (score of AC24).

$\text{DRDscore}=\sum_{}^{}\text{regression coefficient}\left({\text{Feature}}_{\text{i}}\right)\times \text{ score}({\text{Feature}}_{\text{i}})$.

### Assessment of the DRDscore Accuracy in Predicting Platinum Sensitivity

**Figure 4:**
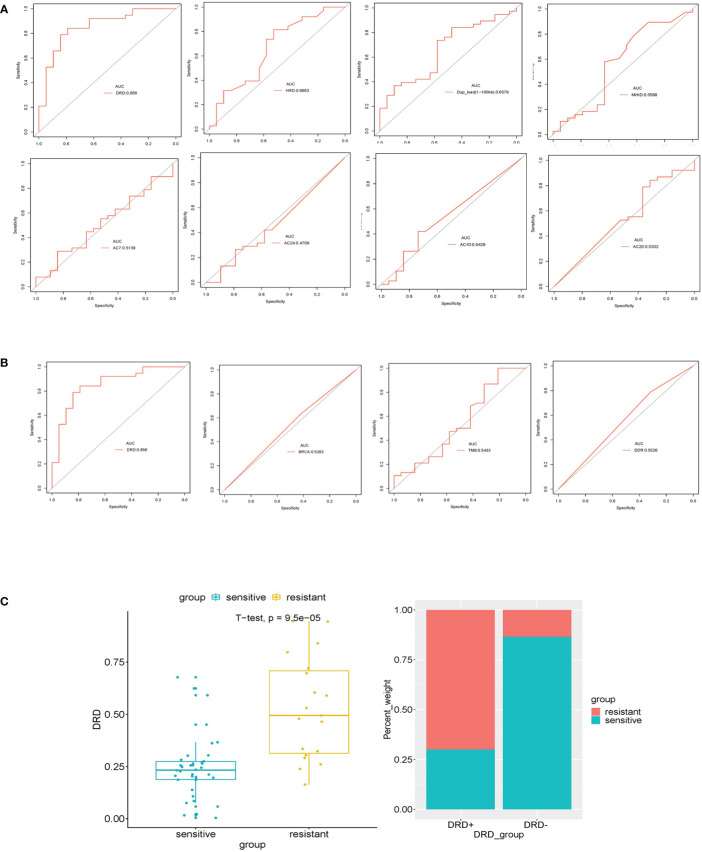
Assessed the Accuracy of DRDscore in prediction of platinum sensitivity in HGSOC patients. **(A)** Analysis of the prediction accuracy between DRDscore and its components with high weight coefficient. **(B)** Analysis of the prediction accuracy between DRDscore and other different candidate features, which included TMB, BRCA and the HRD score with high weight coefficient. **(C)** The difference of the DRDscore value and its prevalence between platinum-sensitive and resistant HGSOC patients.

To evaluate predictive performance, we calculated the area under the receiver operating characteristic curve (ROC) curves of different candidate features, and the DRDscore has outperformed any involved parameters in predicting platinum sensitivity (**Figure 4A**). Meanwhile, our results found that AUC values of DRDscore, HRDscore, *BRCA1/2* alteration, and DDR alteration were 0.856, 0.666, 0.5263, and 0.553, respectively, indicating that DRDscore outperformed than the previous suggested platinum-related biomarkers in HGSOC (**Figure 4B**). The distributions of DRDscores and other parameters between platinum-sensitive and platinum-resistant subgroups were shown as box plots. The DRD scores were significantly distinct between the platinum-resistant and sensitive patients (p=0.000095) (**Figure 4C**). The threshold of 0.7584 had the highest specificity, so it was chosen as the cutoff value.

### *BRCA1/2* Alterations and DRDscore

**Figure 5:**
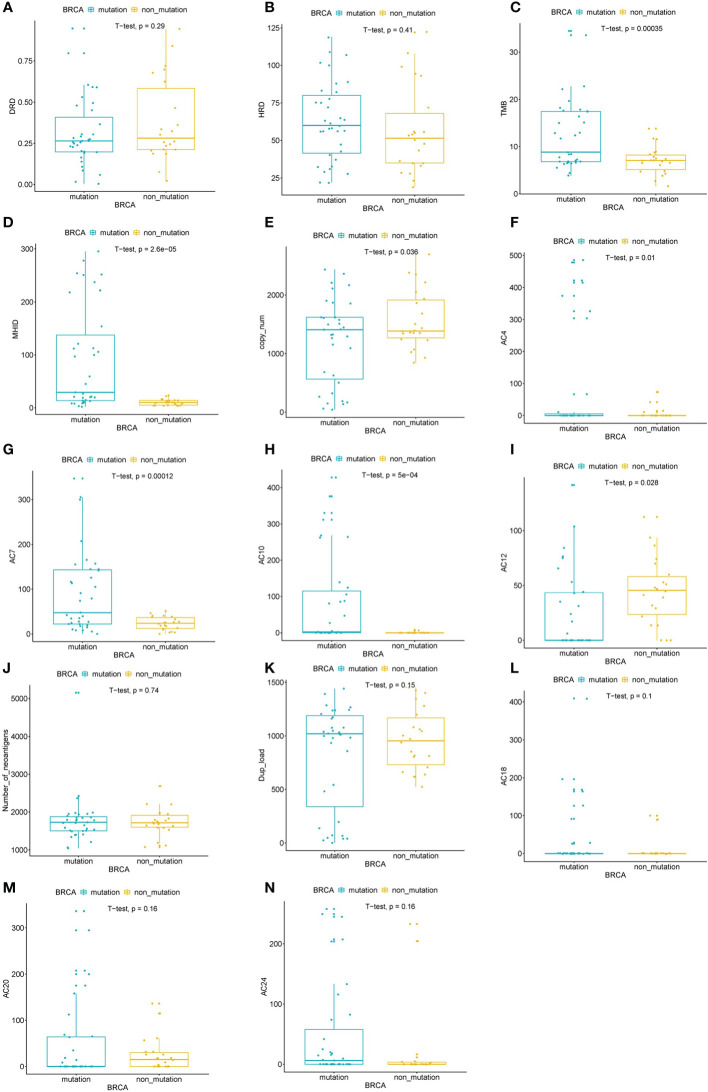
Assessed the correlation between of BRCA alterations and DRDscore. Significant differences of DRDscore **(A)**, HRD score **(B)**, TMB **(C)**, MHID **(D)**, copy number changes **(E)**, mutational signatures, including AC4 **(F)**, AC7 **(G)**, AC10 **(H)**, and AC12 **(I)** between BRCA altered and wildtype HGSOC patients. Analysis of the numbers of neoantigens **(J)**, duplication load of 1–100 kb **(K)**, mutational signatures, including AC18 **(L)**, AC20 **(M),** and AC24 **(N)** between BRCA altered and wildtype HGSOC patients.

The DRDscore and the mainly parameter HRDscore were not significantly correlated to the *BRCA1/2* alteration status in this study (**Figures 5A, B** **)**. However, parameters including TMB value, MHID number, copy number changes, AC4, AC7, AC10, and AC12 were significantly different between *BRCA1/2* altered and wildtype HGSOC patients (**Figures 5C–I**). Other parameters, including neoantigen numbers, duplication of 1 to 100 kb load, mutational signature AC18, AC20 and AC24 were not significantly differed between *BRCA1/2* mutated and wildtype HGSOC (**Figures 5J–N**).

### Predictive Performance in Validation Cohort

**Figure 6:**
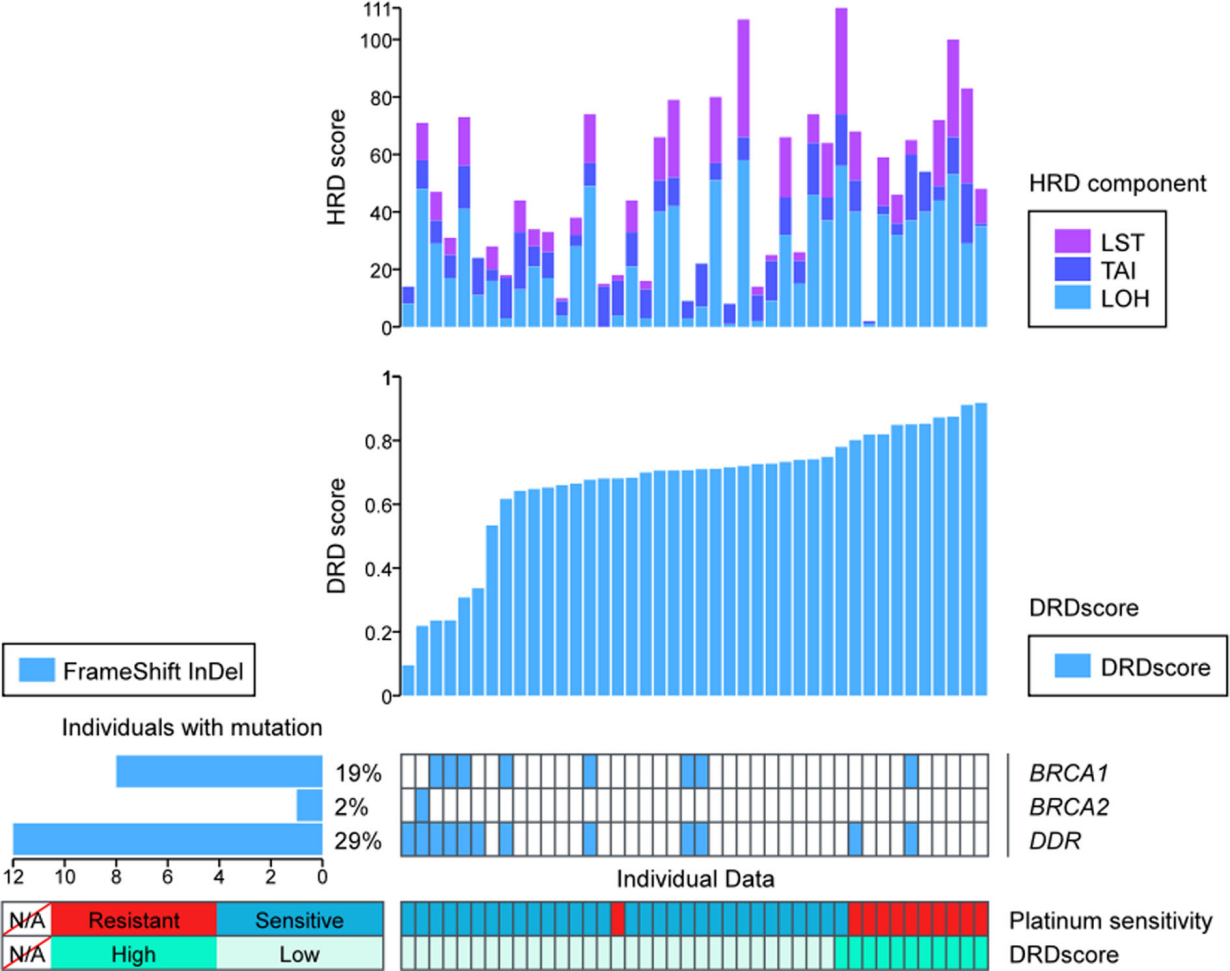
Assessed the correlation between of DRDscore and platinum-resistance in validation cohort.

The validation cohort consisted of eleven platinum-resistant and thirty-one sensitive patients. **Figure 6** showed the prevalence of platinum-resistant in patients with high or low DRDscores. 90.9% (10/11) of platinum-resistant patients had a DRDscore above 0.7584. Only 1 (3.23%) of the platinum-sensitive patients had a high DRDscore. The sensitivity, specificity, and accuracy of predicting platinum-resistance in this cohort was 90.91% (95% CI, 58.72%–99.77%), 96.77% (95% CI, 83.30%–99.92%), and 95.24% (95% CI, 83.84%–99.42%), respectively.

## Discussion

Although HGSOC is one of the most common epithelial ovarian cancers, the composition of mutation types and their correlation with chemotherapy response remain poorly understood. Our study performed a regression analysis based on multiple factors related to DNA damage, including HRD, MHID, TMB, and the number of neoantigen, to determine the factors related to platinum sensitivity and fit into the formula defined as DRDscore.

Accumulating evidence indicates that genetic alterations are prone to bring out the alterations of anti-apoptosis signals, thereby leading to the aberrant responses to chemotherapy (30, 31). Various genetic alterations have been demonstrated to be involved in the primary chemoresistance incidence (32, 33). However, no significant difference in the prevalence or mutational types of genes were found between platinum sensitive and resistant HGSOC patients. Given the curial role of aberrant DNA damage repair in platinum-based chemo-resistance development, we further analyzed differentially mutations of DNA damage repair associated genes in the two groups. No specifically higher prevalence of DDR genes, even including *BRCA1/2* was identified in the platinum-sensitivity patients. Though many studies have suggested the correlation between *BRCA1/2* alterations and platinum-sensitivity, there was evidence that whether having LOH in *BRCA1/2* locus may further determine the platinum response in HGSOC patients with germline *BRCA1/2* variants (34). Meanwhile, *BRCA1/2* alterations and HRD have been widely investigated in the platinum-sensitive HGSOS patients, serving as the biomarkers not only for the sensitivity of PARP inhibitors but also for the surgery selection and prognosis (35). Past studies have found secondary cytoreductive surgery (SCS) could significantly prolong time to progression both in isolated platinum-sensitive or resistant recurrent ovarian cancer patients, and *BRCA1/2* status could also serve as an effective biomarker to select patients who may benefit from SCS, indicating by the significant improve in the progression-free survival than the wildtype patients (36, 37). However, the *BRCA1/2* status was not associated with the clinical benefit for salvage lymphadenectomy in recurrent ovarian cancer patients with isolated lymph-node recurrence, demanding further validation in more and larger studies (38).

Increasing evidence implicates that diverse factors might participate in the platinum response, including the genetic mutation, expression of neoantigen and so on. HRD score is the core parameter for this predictor, as it has been previously proved significantly associated with platinum sensitivity in ovarian cancer and has the highest weight in multivariate regression in our study (39). Though less significantly than the HRDscore, TMB (40), MHID (41), copy number change load (42), has also been indicated as the potential predictor of platinum sensitivity. The other involved parameters in DRDscore, including duplication of 1 to 100 kb and other mutational signatures, have not been reported about their association with the platinum sensitivity before, but have a contribution for the accuracy in our model after multivariate regression. Thus, we further designed DRDscore using LASSO regression with HRD Score, mutational signatures, MHID, and neoantigen number as the basic parameters.

Specific genes mutations, such as p53, and systemic TMB evaluation in several tumor types had been elucidated and served as potential indicators for drugs resistance incidence (43). NJ Birkbak and his colleagues provided evidence to suggest that tumor mutation burden analysis could forecast the outcome in ovarian cancer with *BRCA1* or *BRCA2* mutations (44). Our findings further figure out several factors related to DNA damage, including HRD, MHID, TMB, and neoantigen that participate in the platinum resistance development, to design multiple factors based on DRDscore. The DRDscore established using LASSO regression revealed high accuracy and reliability compared to previous TMB, HRD or MHID score by ROC, providing improved guidance for tumor analysis and diagnosis research. Because all tumor tissues we have collected were archived, they were invalid for RNA sequencing. Though the accuracy of our study outperformed than the previous report (20), a combination of genetic and expression data would further improve the prediction accuracy in future.

There were still some limitations in our current research. Firstly, the sample size in our study remains small and a larger subgroup for more sequencing results need to be addressed. Secondly, we focused on the gene mutation level, and further potential events of alterations in protein level such as protein translation or ubiquitination are also considerable in tumor progression.

In conclusion, we established and evaluated the prognostic value of an innovative score system, which might serve as a potential predictor for platinum resistance in HGSOC. Our study suggested that the model is tightly correlated with clinical platinum resistant characteristics, which reveal improved accuracy in predicting platinum resistance. More importantly, our data described potential to guide personalized therapy and explore new biomarkers for platinum resistance in HGSOC.

## Data Availability Statement

The raw sequencing data files were deposited in the Chinese National Genomics Data Center (https://bigd.big.ac.cn/). Left biospecimens may be shared for academic research under a prior approval from the government of China.

## Ethics Statement

The studies involving human participants were reviewed and approved by Ethics Committee of Peking University Third Hospital (2019-409-1). The patients/participants provided their written informed consent to participate in this study.

## Author Contributions

YL: data collection and drafting the article. XZ: data collection and analysis. YG: data collection. CS: data collection. BY: data collection. TW: data collection. JS: genomic testing results analysis. KH: data collection. CHu: data collection. YW: design of this work and data analysis. HG: design of this work. CHa: design of this work and revise the manuscript. All authors contributed to the article and approved the submitted version.

## Funding

This work was supported by the Fund for Fostering Young Scholars of Peking University Health Science Center (BMU2020PYB030) and Ningxia Key Research and Development Program (2019BFG02002).

## Conflict of Interest

JS was employed by Lifehealthcare Clinical Laboratories.

The remaining authors declare that the research was conducted in the absence of any commercial or financial relationships that could be construed as a potential conflict of interest.
